# MOG encephalomyelitis: international recommendations on diagnosis and antibody testing

**DOI:** 10.1186/s12974-018-1144-2

**Published:** 2018-05-03

**Authors:** S. Jarius, F. Paul, O. Aktas, N. Asgari, R. C. Dale, J. de Seze, D. Franciotta, K. Fujihara, A. Jacob, H. J. Kim, I. Kleiter, T. Kümpfel, M. Levy, J. Palace, K. Ruprecht, A. Saiz, C. Trebst, B. G. Weinshenker, B. Wildemann

**Affiliations:** 1 Molecular Neuroimmunology Group, Department of Neurology, University Hospital Heidelberg, Im Neuenheimer Feld 350, 69120 Heidelberg, Germany; 2 Department of Neurology and Clinical and Experimental Multiple Sclerosis Research Center, Charité – Universitätsmedizin Berlin, Berlin, Germany; 3 NeuroCure Clinical Research Center and Clinical and Experimental Multiple Sclerosis Research Center, Berlin, Germany; 4 Department of Neurology, University of Düsseldorf, Düsseldorf, Germany; 5 Department of Neurology, University of Southern Denmark, Odense, Denmark; 6 Children’s Hospital at Westmead, University of Sydney, Sydney, Australia; 7 Department of Neurology, Hôpital de Hautepierre, Strasbourg Cedex, France; 8 IRCCS, National Neurological Institute C. Mondino, Pavia, Italy; 9 Department of Multiple Sclerosis Therapeutics, Tohoku University Graduate School of Medicine, Sendai, Japan; 10 The Walton Centre, Walton Centre NHS Foundation Trust, Liverpool, UK; 11 Department of Neurology, Research Institute and Hospital of National Cancer Center, Goyang, South Korea; 12 Department of Neurology, Ruhr University Bochum, Bochum, Germany; 13 Institute of Clinical Neuroimmunology, Ludwig Maximilian University, Munich, Germany; 14 Department of Neurology, Johns Hopkins Hospital, Cleveland, USA; 15 Nuffield Department of Clinical Neurosciences, John Radcliffe Hospital, Oxford, UK; 16 Department of Neurology, Charité – Universitätsmedizin Berlin, Berlin, Germany; 17 Service of Neurology, Hospital Clinic, and Institut d’Investigacions Biomèdiques August Pi i Sunyer (IDIBAPS), Universitat de Barcelona, Barcelona, Spain; 18 Department of Neurology, Hannover Medical School, Hanover, Germany; 19 Department of Neurology, Mayo Clinic, Rochester, MN USA

**Keywords:** Myelin oligodendrocyte glycoprotein (MOG) antibodies, Consensus recommendations, Diagnosis, Antibody testing, Multiple sclerosis (MS), Neuromyelitis optica spectrum disorders (NMOSD), Optic neuritis (ON), Myelitis

## Abstract

Over the past few years, new-generation cell-based assays have demonstrated a robust association of autoantibodies to full-length human myelin oligodendrocyte glycoprotein (MOG-IgG) with (mostly recurrent) optic neuritis, myelitis and brainstem encephalitis, as well as with acute disseminated encephalomyelitis (ADEM)-like presentations. Most experts now consider MOG-IgG-associated encephalomyelitis (MOG-EM) a disease entity in its own right, immunopathogenetically distinct from both classic multiple sclerosis (MS) and aquaporin-4 (AQP4)-IgG-positive neuromyelitis optica spectrum disorders (NMOSD). Owing to a substantial overlap in clinicoradiological presentation, MOG-EM was often unwittingly misdiagnosed as MS in the past. Accordingly, increasing numbers of patients with suspected or established MS are currently being tested for MOG-IgG. However, screening of large unselected cohorts for rare biomarkers can significantly reduce the positive predictive value of a test. To lessen the hazard of overdiagnosing MOG-EM, which may lead to inappropriate treatment, more selective criteria for MOG-IgG testing are urgently needed. In this paper, we propose indications for MOG-IgG testing based on expert consensus. In addition, we give a list of conditions atypical for MOG-EM (“red flags”) that should prompt physicians to challenge a positive MOG-IgG test result. Finally, we provide recommendations regarding assay methodology, specimen sampling and data interpretation.

## Background

Over the past few years, the role of immunoglobulin G serum antibodies to myelin oligodendrocyte glycoprotein (MOG-IgG) in patients with inflammatory CNS demyelination has been revisited. While antibodies to MOG were originally thought to be involved in multiple sclerosis (MS), based on results from enzyme-linked immunosorbent assays employing linearized or denatured MOG peptides as antigen, more recent studies using new-generation cell-based assays have demonstrated a robust association of antibodies to full-length, conformationally intact human MOG protein with (mostly recurrent) optic neuritis (ON), myelitis and brainstem encephalitis, as well as with acute disseminated encephalomyelitis (ADEM)-like presentations, rather than with classic MS [1–11].

Based on evidence from (a) immunological studies suggesting a direct pathogenic impact of MOG-IgG, (b) neuropathological studies demonstrating discrete histopathological features, (c) serological studies reporting a lack of aquaporin-4 (AQP4)-IgG in almost all MOG-IgG-positive patients, and (d) cohort studies suggesting differences in clinical and paraclinical presentation, treatment response and prognosis, MOG-IgG is now considered to denote a disease entity in its own right, distinct from classic MS and from AQP4-IgG-positive neuromyelitis optica spectrum disorders (NMOSD), which is now often referred to as MOG-IgG-associated encephalomyelitis (MOG-EM) [11–13].

Importantly, however, MOG-EM and MS show a relevant phenotypic, i.e., clinical as well as radiological, overlap [3, 14]: like MS, MOG-EM follows a relapsing course in most cases [3, 6], at least in adults, and 33 and 15% of adult patients with MOG-EM meet McDonald’s and Barkhof’s criteria for MS, respectively, at least once over the course of disease [3, 14]. Accordingly, many patients with MOG-EM were falsely classified as having MS in the past [3, 4]. However, such misclassification has potential therapeutic implications: (a) similar to what has been observed in AQP4-IgG-positive NMOSD, some drugs approved for MS might be ineffective or even harmful in MOG-EM owing to differences in immunopathogenesis [3, 4, 15–17]; (b) MOG-EM is associated with a high risk of flare-ups after cessation of steroid treatment for acute attacks and may thus require close monitoring and careful steroid tapering [3, 18–22]; and (c) patients positive for MOG-IgG might be particularly responsive to antibody-depleting treatments for acute attacks such as plasma exchange or immunoadsorption [3, 4, 9, 14, 23, 24], to B cell-targeted long-term therapies such as rituximab, to treatment with intravenous immunoglobulins (IVIG) (especially in children [25]), and to immunosuppressive treatments [3, 6, 14, 25, 26]. Therefore, increasing numbers of patients with suspected or established MS are currently being screened for MOG-IgG.

However, screening of large unselected populations for rare biomarkers generally decreases the positive predictive value of diagnostic tests by increasing the rate of false-positive results [27, 28]. Even if assays with high specificity (≥99%) are used, true-positive (TP) results can easily be outnumbered by false-positive (FP) results if the prevalence of a marker is low and the number of samples tested is high. This also applies to MOG-IgG testing. Based on a hypothetical prevalence of 1% genuinely MOG-IgG-positive cases among all patients currently diagnosed with MS, testing of 100,000 patients with an almost flawless, 99% specific and 100% sensitive assay would result in an unacceptable ratio of 990 FP results to 1000 TP results. Therefore, unselected screening of all patients with suspected or established MS for MOG-IgG should be discouraged and more specific criteria for MOG-IgG testing are urgently needed.

In this paper, we propose for the first time indications for MOG-IgG testing based on expert consensus. In addition, we give a list of conditions considered atypical for MOG-EM (“red flags”) that should prompt physicians to challenge the validity of a positive MOG-IgG test result. Finally, we provide recommendations regarding assay methodology, specimen sampling, and data interpretation.

## Methods

PubMed was searched for articles published between February 2007 and February 2017 using the following search term: (“myelin oligodendrocyte glycoprotein” OR MOG) AND (antibody OR antibodies OR IgG). All articles identified by this means were reviewed by a core group of physicians (S.J., B.W., F.P., K.R.) for clinical and paraclinical findings that have been frequently reported in association with MOG-IgG seropositivity in patients with CNS demyelination and which, therefore, may justify MOG-IgG testing, as well as for potential “red flags”, i.e., conditions that are typically found in inflammatory disorders of the CNS but have been reported to be absent or very rare in MOG-IgG-positive patients and thus may indicate diagnoses other than MOG-EM. Based on core group consensus, a first set of recommendations was formulated and then circulated to a broader panel of experts in the field from Australia, Denmark, France, Germany, Italy, Japan, South Korea, Spain, UK, and the USA for discussion and refinement. Panel members were invited by the core group based on eminence and previous contributions to the field. Based on several rounds of core group-led peer-to-peer discussions of the individual recommendations with all individual members of the panel, a final set of evidence- as well as eminence-based recommendations was drawn up to which all members gave their approval. All recommendations given here should be considered as expert consensus.

## Recommendations on MOG-IgG testing

In Table 1, we propose indications for MOG-IgG testing based on clinical and paraclinical findings that are typical of MOG-EM and/or atypical for MS and were considered by the panel members to be associated with pre-test odds high enough to justify MOG-IgG testing or that demand MOG-IgG testing because of potentially significant therapeutic consequences of a positive test result according to expert consensus. These recommendations apply to all patients with suspected CNS demyelination of putative autoimmune etiology and an either monophasic or relapsing disease course. Given the very low pre-test probability [29], we recommend against general MOG-IgG testing in patients with a progressive disease course. In Table 2, we give a number of case vignettes of patients considered to be at high risk of MOG-EM to illustrate the broad spectrum of symptoms associated with that syndrome and the practical feasibility and relevance of the proposed criteria. In Table 3, we give a number of recommendations regarding assay selection, specimen sampling, and data interpretation. Finally, Table 4 lists conditions (“red flags”) that we believe are atypical for MOG-EM and should thus prompt physicians to challenge a positive MOG-IgG test result and seek a better explanation for the patients’ clinical and paraclinical findings.

**Table 1:** Recommended indications for MOG-IgG testing in patients presenting with acute CNS demyelination of putative autoimmune etiology

1. Monophasic or relapsing acute optic neuritis, myelitis, brainstem encephalitis, encephalitis, or any combination thereof, *and* 2. radiological or, only in patients with a history of optic neuritis, electrophysiological (VEP) findings compatible with CNS demyelination, *and* 3. at least one of the following findings: *MRI* a. Longitudinally extensive spinal cord lesion (≥3 VS, contiguous) on MRI (so-called LETM)^a,b^ b. Longitudinally extensive spinal cord atrophy (≥3 VS, contiguous) on MRI in patients with a history compatible with acute myelitis^a^ c. Conus medullaris lesions, especially if present at onset^c^ d. Longitudinally extensive optic nerve lesion (e.g., >1/2 of the length of the pre-chiasmal optic nerve, T2 or T1/Gd)^d^ e. Perioptic Gd enhancement during acute ON^e^ f. Normal supratentorial MRI in patients with acute ON, myelitis and/or brainstem encephalitis g. Brain MRI abnormal but no lesion adjacent to a lateral ventricle that is ovoid/round or associated with an inferior temporal lobe lesion and no Dawson’s finger-type or juxtacortical U fiber lesion (Matthews-Jurynczyk criteria^f^) h. Large, confluent T2 brain lesions suggestive of ADEM *Fundoscopy* i. Prominent papilledema/papillitis/optic disc swelling during acute ON *CSF* j. Neutrophilic CSF pleocytosis^g^or CSF WCC > 50/μl^h^ k. No CSF-restricted OCB as detected by IEF at first or any follow-up examination^i^ (applies to continental European patients only) *Histopathology* l. Primary demyelination with intralesional complement and IgG deposits m. Previous diagnosis of “pattern II MS” ^j^ *Clinical findings* n. Simultaneous bilateral acute ON o. Unusually high ON frequency or disease mainly characterized by recurrent ON p. Particularly severe visual deficit/blindness in one or both eyes during or after acute ON q. Particularly severe or frequent episodes of acute myelitis or brainstem encephalitis r. Permanent sphincter and/or erectile disorder after myelitis s. Patients diagnosed with “ADEM”, “recurrent ADEM”, “multiphasic ADEM” or “ADEM-ON” t. Acute respiratory insufficiency, disturbance of consciousness, behavioral changes, or epileptic seizures (radiological signs of demyelination required) u. Disease started within 4 days to ~ 4 weeks after vaccination v. Otherwise unexplained intractable nausea and vomiting or intractable hiccups (compatible with area postrema syndrome)^a^ w. Co-existing teratoma or NMDAR encephalitis (low evidence^k^) *Treatment response* x. Frequent flare-ups after IVMP, or steroid-dependent symptoms^l^ (including CRION) y. Clear increase in relapse rate following treatment with IFN-beta or natalizumab in patients diagnosed with MS (low evidence)	

Note that these recommendations are primarily intended for use in adults and adolescents. Indications for MOG-IgG testing in young children need not to be as rigorous as in adults, since MOG-EM is thought to be significantly more frequent among young children with acquired demyelinating disease (up to 70%; frequency declining with age) than among their adult counterparts (≤ 1% in Western countries; probably ≤ 5% in Japan and other Asian countries because of lower MS prevalence), which reduces the risks attached to antibody screening outlined in the *Introduction*

Abbreviations: *ADEM* acute disseminated encephalomyelitis, *ADEM-ON* ADEM with recurrent ON, *AQP4* aquaporin-4, *CNS* central nervous system, *CRION* chronic relapsing inflammatory optic neuropathy, *CSF* cerebrospinal fluid, *EM* encephalomyelitis, *Gd* gadolinium, *IA* immunoadsorption, *IgG* immunoglobulin G, *IVMP* intravenous methylprednisolone, *LE* left eye, *LETM* longitudinally extensive transverse myelitis, *MOG* myelin oligodendrocyte glycoprotein, *MRI* magnetic resonance imaging, *MS* multiple sclerosis, *NMDAR* N-methyl-D-aspartate receptor, *NMO* neuromyelitis optica, *OCB* oligoclonal IgG bands, *ON* optic neuritis, *PEX* plasma exchange, *RE* right eye, *RRMS* relapsing-remitting MS, *VEP* visual evoked potentials, *VS* vertebral segments, *WCC* white cell count

^a^If costs play a role and disease is stable: test AQP4-IgG first, since more frequent in that condition than MOG-IgG. If disease is active, requiring fast decision-making, or if costs play no role: test AQP4-IgG and MOG-IgG in parallel

^b^LETM is common both in MOG-EM and in AQP4-NMOSD, but rarely if ever occurs in MS; as a caveat, however, non-contiguous lesions may mimic LETM in some patients with MS. N.B.: Short lesions do not per se exclude MOG-EM. MRI shows short lesions at least once over the disease course in around 44–52% of all MOG-EM patients [3, 39] and around 15% of all AQP4-NMOSD patients [40]. Lesion length may also depend on MRI timing issues, with shorter lesions detected when the MRI was performed early in the evolution of acute myelitis or in clinical remission. Both axial and sagittal plane images should be used to judge lesion extent. LETM has also been shown to be frequently present at disease onset in MOG-IgG-positive children with manifestations other than isolated ON (32/40 or 80% of all examined cases) [41]

^c^Present in 6/8 patients in [7] (at onset); 4/6 in [8]; 4/11 in [35]; 3/12 in [42]; 5/26 (not all had lumbar MRI) in [3]; and in 13/40 pediatric patients (at onset) with manifestations other than isolated ON [41]

^d^Ramanathan et al. (2015) reported a median optic nerve lesion length of 23.1 mm (IQR 18-33) in MOG-IgG-related ON (N=19); this compares to a median lesion length of 9.9 mm (IQR 6.6-19.8; N=13) in MS-related ON observed in the same study [43] and of 10.5 mm in a second, independent study (N=26) [44]. Recent data suggest that also involvement (T2, T1/Gd or optic nerve swelling) of > 6/12 optic nerve segments (anterior orbital RE/LE, posterior orbital RE/LE, canalicular RE/LE, intracranial RE/LE, chiasm right/left half, optic tract right/left side) may be associated with increased pre-test odds for MOG-IgG (observed in 6/19 [32%] MOG-IgG-positive ON patients but in none of 13 [0%] MS-ON patients) [43]. Longitudinal extensive lesions involving at least 4 of 5 segments (anterior intraorbital segment, posterior intraorbital segment, canalicular, intracranial, chiasmal) were also noted in >=50% of MOG-IgG-positive patients in [45]. By contrast, lesions in MS-related ON extended only over 1 (70%) or 2 (30%) of 9 segments (intraorbital RE/LE, canalicular RE/LE, intracranial RE/LE, chiasmal, optic tract right/left side) in [44], and a mean extension of just 2.2/10 segments (orbital RE/LE, canalicular RE/LE, intracranial RE/LE, chiasma right/left half, optic tract right/left side) was observed in MS-ON in [46]. Longitudinal extensive lesions ranging over more than the half of the distance between the optic nerve head and the chiasm were also reported in 3/3 patients in [47] and in 6/10 (60%) in [3]. Finally, 9/10 MOG-ON Han patients (90%) showed involvement of all three segments of the pre-chiasmal optic nerve (intraorbital, canalicular, intracranial) in [48], in 6 of whom chiasmal and/or optic tract involvement was noted in addition

^e^Observed in 11/28 patients during acute ON in [3], in 6/18 in [49], and in 6/8 in [48], but not usually in MS. Perioptic T2 hyperintensity alone does not count

^f^Positive in ≥ 90% of RRMS patients [37, 36, 50]. By contrast, ovoid/round lesions adjacent to a lateral ventricle, lesions adjacent to a lateral ventricle in association with a temporal lobe lesion, and Dawson’s finger-type lesions were absent in 21/21 (100%) MOG-IgG-positive patients in a mixed adult (*n* = 15) and pediatric (*n* = 6) cohort [36, 37] and juxtacortical U fiber lesions in 20/21 (95.2%). Recently, a lack of Dawson’s finger-type lesions in MOG-IgG-positive patients has been confirmed in an exclusively pediatric cohort (absent in 68/69 [98.6%]; the only patient positive for Dawson’s finger lesions had typical MS and was negative for MOG-IgG at re-testing); U fiber lesions were absent in 65/69 (94.2%) MOG-IgG-positive pediatric patients in the same study [41]

^g^Present at least once in 64% of patients with pleocytosis [3] (median 22% of all white cells; range 3–69%) but typically absent in MS. N.B.: Neutrophilic pleocytosis is also frequently found in AQP4-IgG-positive NMOSD [51]

^h^Observed in 43% (14/36) of MOG-IgG-positive patients with pleocytosis (peak values) [3], but only rarely in patients with MS (≤ 2% according to [52]; 1/71 patient ≥ 15 years of age [range 15–29] in [53])

^i^Oligoclonal bands (OCB) have been reported in up to 98% of patients with MS in central and Northern Europe [53] but only in around 12–13% of patients with MOG-EM in two recent Central European studies [3, 54]; of note, many MOG-EM patients previously falsely diagnosed with MS were atypical in that they had no OCB in a recent multicenter study [3]. As a caveat, it should be noted that positive OCB do NOT exclude MOG-EM [3] and that the frequency of OCB in MS may be lower in Asian patients (e.g., 40–80% in Japan) as well as in some regions in Europe such as Sardinia (84% in a recent study [55]), possibly depending on genetic factors. “No CSF-restricted OCB” refers to the presence of OCB patterns 1 (no OCB), 4 (mirror pattern without additional IgG bands present exclusively in the CSF), or 5 (monoclonal IgG band present both in the CSF and in the serum) [56]

^j^Some patients diagnosed with “pattern II MS” lesions, which are characterized by IgG and complement deposits, were shown by independent groups to have in fact MOG-EM, suggesting that the current histopathological criteria may not be sufficiently specific to distinguish between MS and MOG-EM [24, 57, 58]

^k^Patients with teratoma and positive MOG-IgG serostatus have been identified in two cohorts so far (2/74; 3%) [3, 4, 59]; expression of CNPase, an oligodendrocyte marker, has been described in mature teratomas, and oligodendrogliomas may arise in mature teratomas. Additional testing for NMDAR antibodies is highly recommended in patients with teratoma and neurological symptoms [60]. Recent, though preliminary, reports suggest that MOG-EM and NMDAR encephalitis may occasionally co-exist [61]

^l^Re-occurrence of symptoms after tapering of oral steroids [3, 18, 20, 22, 62]

**Table 2:** Case vignettes of patients at risk of MOG-IgG seropositivity (examples)

Example 1: 35-year-old woman presenting with bilateral acute ON. Develops transient blindness; fundoscopy shows papilledema; lumbar puncture reveals lymphomonocytic pleocytosis with 10% neutrophils and negative OCBs; brain MRI shows perioptic Gd enhancement but is otherwise normal; flaring up of symptoms after tapering of oral steroids; later recurrent ON attacks, stabilization with rituximab. Example 2: 40-year-old woman with two attacks of acute, OCB-negative myelitis. Spine MRI shows an isolated short spinal cord lesion at first attack and a longitudinally extensive spinal cord lesion at relapse; brain MRI abnormal but no Dawson’s finger-type lesion, no juxtacortical U fibre lesion, and no lesion adjacent to a lateral ventricle that is ovoid or associated with an inferior temporal lobe lesion [36, 37, 50]; flaring up of myelitis symptoms after discontinuation of intravenous steroid treatment, good response to PEX. Example 3: Young man with a previous diagnosis of “OCB-negative RRMS”. Predominantly ON and myelitis attacks; conus lesion with severe erectile and sphincter disturbance after first myelitis; longitudinally extensive optic nerve lesion with involvement of the optic chiasm; increase in relapse rate under treatment with interferon-beta but stabilization with rituximab. Example 4: 42-year-old woman presenting with incomplete, painful tetraparesis. Previous diagnosis of RRMS with positive OCB; spinal cord MRI reveals a contiguous lesion extending from C3 to T1; negative serology for AQP4-IgG. Example 5: ADEM-like presentation with large white matter lesions and disturbance of consciousness, brainstem lesions, and involvement of the entire spinal cord in a 25-year-old woman; onset 3 weeks after vaccination. Example 6: Simultaneous unilateral ON and LETM extending into the brainstem in a 39-year-old man. CSF pleocytosis (90 white cells/μl) with 5% neutrophils; no CSF-restricted OCB; negative AQP4-IgG serostatus. Example 7: Young woman presenting with recurrent and steroid-dependent isolated ON, previously classified as CRION; normal brain MRI. Example 8: Young man with acute encephalitis and seizures. MRI reveals large cortical/subcortical white matter lesions not involving the inferior temporal lobe; good response to steroids; negative for typical viral and autoimmune causes of encephalitis.	

Abbreviations: *ADEM* acute disseminated encephalomyelitis, *AQP4* aquaporin-4, *CRION* chronic relapsing inflammatory optic neuropathy, *CSF* cerebrospinal fluid, *EM* encephalomyelitis, *Gd* gadolinium, *IgG* immunoglobulin G, *LETM* longitudinally extensive transverse myelitis, *MOG* myelin oligodendrocyte glycoprotein, *MRI* magnetic resonance tomography, MS multiple sclerosis, *ON* optic neuritis, RRMS relapsing-remitting MS

**Table 3:** Recommendations on methodology, test parameters, specimen sampling and data interpretation

Assay types *Cell-based assays (IFT/FACS):* Recommended (current gold standard); must employ full-length human MOG as target antigen; use of Fc-specific (or IgG1-specific [63]) secondary antibodies highly recommended to avoid cross-reactivity with (specifically or non-specifically co-binding) IgM and IgA antibodies [11, 63] *Immunohistochemistry:* Currently not recommended (less sensitive than cell-based assays, limited data available on specificity [11, 64], sensitivity depends on tissue donor species [64]); if used, Fc-specific secondary antibodies adsorbed against tissue donor IgG required in order to avoid cross-reactivity with IgM and IgA or with tissue-bound donor IgG *Peptide-based ELISA, Western blot:* Insufficiently specific, obsolete Biomaterial *Serum:* Recommended (specimen of choice); shipment at 4 °C or on dry ice advisable if samples do no arrive within 1–2 days *Cerebrospinal fluid:* Not usually required, since MOG-IgG is produced mostly extrathecally, resulting in lower CSF than serum titers [2]; potentially helpful in rare, selected cases (e.g., strong background due to co-existing high-titer non-MOG serum antibodies); shipment at 4 °C or on dry ice advisable Immunoglobulin classes *Testing for MOG-IgG:* Recommended *Testing for MOG-IgM and/or MOG-IgA:* Currently not recommended; additional MOG-IgM and MOG-IgA antibodies have been described in some MOG-IgG-positive patients [1, 2]; the clinical relevance of isolated MOG-IgM or -IgA results is unknown; testing for antibodies of the IgM class requires removal of total IgG from the sample to avoid both false-negative (due to high-affinity IgG displacing IgM) and false-positive (due to IgM anti-IgG_Fc_ rheumatoid factors) results [65] Data reporting Immunoglobulin class detected, assay type, antigenic substrate and biomaterial used, titer/concentration/units, assay-specific cut-offs and performing laboratory should all be documented (e.g., “Serum MOG-IgG 1:1280 [cut-off ≥ 1:160^a^; assay: live CBA, Innsbruck lab; antigen: full-length human MOG]”) Data interpretation As with all laboratory tests, positive test results should always be interpreted in the context of the patient’s overall presentation; if “red flags” as defined in Table 4 are present, re-testing of the positive serum sample (or, if not anymore available, at least testing of a follow-up serum sample) is recommended; to reduce the potential risk of reproducing false-positive results due to issues inherent to the very method employed, use of a second (and, in the case of discrepant results, third) methodologically different cell-based assay is advisable; if in doubt, seek expert advice from a specialized center Timing issues MOG-IgG serum concentrations depend on disease activity (with higher median concentrations during acute attacks than during remission) and treatment status (with lower concentrations while on immunosuppression) and may transiently vanish after plasma exchange [3]; if MOG-IgG is negative but MOG-EM still suspected, re-testing during acute attacks, during treatment-free intervals, or 1–3 months after plasma exchange (or IVIG^b^) is recommended – N.B.: Some cases of monophasic MOG-positive EM/ADEM in adult patients have been described in which MOG-IgG disappeared permanently following clinical recovery [2–4, 33–35]	

Abbreviations: *ADEM* acute disseminated EM, *CBA* cell-based assay, *CSF* cerebrospinal fluid, *ELISA* enzyme-linked immunosorbent assay, *EM* encephalomyelitis, *FACS* fluorescence-activated cell sorting, *IgG/A/M* immunoglobulin G/A/M, *IFT* indirect fluorescence test, *IVIG* intravenous immunoglobulins, *MOG* myelin oligodendrocyte glycoprotein, *PEX* plasma exchange

^a^Note that the cut-off given here is an example only; actual cut-off values are assay-dependent.

^b^Generally, pretreatment with IVIG is liable to cause false negative and false positive results in antibody assays [66–68]; whether any of the tests currently used for detecting MOG-IgG are affected by IVIG pretreatment has not been investigated so far.

**Table 4:** “Red flags”: conditions that should prompt physicians to challenge a positive test result (consider re-testing the patient, ideally using an alternative, i.e., methodologically different cell-based assay; in case of doubt, consider seeking expert advice from a specialized center)

*Disease course* Chronic progressive disease (very rare in MOG-IgG-positive patients [3]), including SPMS (especially SPMS without relapses) and PPMS^a^ Sudden onset of symptoms, e.g., < 4 h from onset to maximum (consider ischemic cause), or continuous worsening of symptoms over weeks (consider tumor, sarcoidosis, etc.) *MRI* Lesion adjacent to a lateral ventricle that is ovoid/round or associated with an inferior temporal lobe lesion, or Dawson’s finger-type lesion Active brain MRI over time with silent increase in lesion burden between relapses (limited evidence) *CSF* Bi- or trispecific MRZ reaction^b^ (consider MS) *Serology* MOG-IgG levels at or just barely above the assay-specific cut-off^c^, especially (but not exclusively) if clinical picture is atypical Positive MOG-IgM and/or MOG-IgA result with negative MOG-IgG (clinical significance unknown) MOG-IgG positivity in the CSF but not in the serum^d^ (MOG-IgG is typically produced extrathecally) AQP4-IgG/MOG-IgG “double-positive” test results (extremely rare; should prompt retesting for both antibodies)^e^ *Others* Clinical or paraclinical findings suggesting diagnoses other than MOG-EM, NMOSD or MS (e.g., neurotuberculosis, neuroborreliosis, neurosyphilis, neurosarcoidosis, Behçet syndrome, subacute combined degeneration of the spinal cord, Leber’s hereditary optic neuropathy, vasculitis, CNS lymphoma, gliomatosis cerebri, paraneoplastic neurological disorders^f^, PRES, PML, and evidence for CNS infection^g^) Combined central and peripheral demyelination [69] (MOG is not expressed in the peripheral nervous system)^h^	

Abbreviations: *AQP4* aquaporin-4, *CNS* central nervous system, *CSF* cerebrospinal fluid, *EM* encephalomyelitis, *Ig* immunoglobulin, *MOG* myelin oligodendrocyte glycoprotein, *MRZ* measles, rubella and zoster virus, *MS* multiple sclerosis, *NMDAR* N-methyl-D-aspartate receptor, *NMOSD* neuromyelitis optica spectrum disorder, *PPMS* primary progressive MS, *PML* progressive multifocal leukoencephalopathy, *PRES* posterior reversible encephalopathy syndrome, *SPMS* secondary progressive MS, *WCC* white cell count

^a^Just one borderline MOG-IgG result found among 290 patients with PPMS (*n* = 174) or SPMS (*n* = 116) in a recent study [29]

^b^Measles (M), rubella (R), and zoster (Z) reaction: Intrathecal synthesis against at least two of these three viral agents (i.e., against M + R, M + Z, R + Z, or M + R + Z); part of the polyspecific, intrathecal humoral immune reaction in MS; present in around 70% of MS patients but not at all, or only very rarely, in MOG- or AQP4-IgG-positive patients (MOG-EM: 0/11; NMO: 1/42; “ADEM”: 1/26) [3, 70, 71]

^c^Except in patients who were previously positive at levels clearly above the cut-off, in which case low-titer results may reflect true (spontaneous or treatment-related) decline in antibody levels

^d^May be valid in the rare instances in which co-existing serum autoantibodies hamper serum analysis but not CSF analysis (false-negative serum test)

^e^If confirmed in a second assay and IPND criteria for NMOSD are met, co-existence of MOG-EM and AQP4-NMOSD must be assumed

^f^Note, however, that preliminary reports suggest occasional co-incidence of MOG-EM and NMDAR encephalitis [61]; in such patients teratoma needs to be excluded [60]

^g^Note that CSF findings in MOG-EM (as well as in AQP4-NMOSD) may mimic CNS infection with neutrophil pleocytosis, impaired blood-CSF barrier function, and a lack of CSF-restricted oligoclonal bands [3, 40, 51]. White cell counts in MOG-EM ranged between 6 and 306 cells/μl (median 33; quartile range 13–125) in a recent European study [2]; WCC ≥ 100 cells/μl were present at least once in 9/32 (28.1%) patients; neutrophil granulocytes were present at least once in 9/14 (64.3%) patients with pleocytosis and available data (median 22% of all white cells; range 3–69%)

^h^May be true positive in the rare cases in which MOG-EM and unrelated peripheral neuropathy of other cause co-exist

In practice, many patients diagnosed with AQP4-IgG-negative NMOSD according to the IPND 2015 criteria [30] will meet also the criteria for MOG-IgG testing given in Table 1 and should thus be tested. However, MOG-IgG testing should not be restricted to patients with AQP4-IgG-negative NMOSD. While this approach seems to offer simplicity, it would be inappropriate for several reasons: (1) The IPND criteria for AQP4-IgG-negative NMOSD demand dissemination in space, which would prevent testing of many patients with syndromes compatible with MOG-EM (e.g., patients with isolated longitudinally extensive transverse myelitis [LETM], isolated bilateral ON, or isolated brainstem encephalitis). (2) They include magnetic resonance imaging (MRI) criteria that are based on lesion distribution patterns observed in AQP4-IgG-positive NMOSD, some of which reflect areas of high AQP4 expression; however, AQP4 is not the target antigen in MOG-EM. Accordingly, lesion distribution may differ between NMOSD and MOG-EM and some MOG-EM patients do not satisfy these criteria (e.g., patients with recurrent bilateral non-longitudinal ON without chiasm involvement plus non-NMOSD-typical brain lesions, those with severe and recurrent non-longitudinally extensive myelitis, and those with ADEM-like presentation with severe brain and brainstem involvement but no area postrema lesion). (3) Such a recommendation would imply testing of all patients for AQP4-IgG before they were tested for MOG-IgG, which might unnecessarily delay diagnosis and treatment. (4) The criteria for AQP4-IgG-negative NMOSD require exclusion of other diagnoses; this would constitute a logical repugnancy, since a negative test for MOG-IgG would be a prerequisite for MOG-IgG testing. (5) Finally, but less importantly, using NMOSD criteria for diagnosing MOG-EM would, in addition to resulting in a substantial loss in sensitivity and specificity, also be confusing to non-experts, given that AQP4-IgG-positive NMOSD and MOG-EM are distinct diseases with different target antigens (AQP4 vs. MOG), pathophysiology (astrocytopathy vs. primary demyelination), and clinical spectra.

Alternatively, should we restrict MOG-IgG testing to patients with AQP4-IgG-negative NMO according to Wingerchuk’s 2006 criteria [31]? This would again result in a substantial loss of patients at high risk of MOG-EM, since those criteria require a history of both ON and myelitis and would thus be inappropriate. Of note, MOG-IgG testing in patients with seronegative NMO according to the 2006 criteria is already covered by our recommendation to test all patients with LETM for MOG-IgG (see Table 1), since the 2006 criteria strictly require a history of LETM in patients negative for AQP4-IgG.

Instead, we propose to base the indication for MOG-IgG testing in patients with suspected CNS demyelination on the presence of specific clinical and paraclinical findings that are considered typical for MOG-EM and/or atypical for conventional MS (see Table 1).

During the consensus-finding process, concerns were raised regarding inclusion of the following treatment-related indications for MOG-IgG testing in Table 1:

- Particularly good response to antibody-depleting therapies (plasma exchange [PEX], immunoadsorption [IA])
- Particularly good response to B cell-depleting therapies (rituximab, ocrelizumab, ofatumumab) but relapse immediately after re-occurrence of B cells

It was argued by some members of the panel that good responses to PEX, IA, or B cell depletion have also been observed in conventional MS. However, consensus was achieved that if present in addition to any of the indications listed in Table 1, good response to antibody or B cell-depleting treatments or IVIG further increases the pre-test likelihood of MOG-EM and thus supports the decision to test for MOG-IgG.

Taking into account that MOG-IgG serum concentrations depend on disease activity (with higher concentrations during acute attacks) and treatment status (with lower concentrations while on immunosuppression) as well as on assay sensitivity, we recommend re-testing patients during acute attacks or during treatment-free intervals and/or in a second cell-based assay if MOG-IgG was negative at first examination but MOG-EM is still suspected based on the list of indications given in Table 1 [3].

Only sparse data are available on the usefulness of regular monitoring of antibody titers in individual patients known to be positive for MOG-IgG. Median MOG-IgG titers have indeed been shown to be significantly higher during relapse than during remission [2], making regular MOG-IgG testing a potentially promising method for predicting attacks and monitoring treatment efficacy. However, there are several limitations: While titers > 1:2560 were found only during acute attacks in a recent study using a live cell-based assay [2], some patients still had relatively low titers during acute attacks and others had relatively high titers during remission, suggesting that additional factors such as blood–CSF barrier damage, T cell activation, antibody affinity, or complement-activating activity may be involved, with no general cut-off value for relapse induction [2]. In addition, treatment effects could play a role. Finally, intervals sufficient to detect imminent attacks in time have not yet been defined. Based on experience from studies on AQP4-IgG-positive NMOSD, in which serum antibody levels rise only very shortly before an attack [32], very close testing intervals may be required, which would make monitoring both expensive and challenging from a practical point of view. Accordingly, no general recommendation for regular monitoring of MOG-IgG titers for relapse prediction or treatment monitoring can currently be made.

Of note, some patients have been reported in whom MOG-IgG disappeared over time [2, 33–35]. Interestingly, many of these patients had monophasic disease. By contrast, MOG-IgG was detectable at the last follow-up in all patients (*n* = 18) with a relapsing disease course and available follow-up samples (mean interval 33 months since first testing; maximum follow-up period 10 years) in a recent study [2]. Disappearance of MOG-IgG after the initial attack might thus have prognostic implications, and re-testing of MOG-IgG-positive patients 6–12 months after the first attack might therefore be useful. However, there are some limitations: Most of the reported monophasic patients were children or juveniles, and most had ADEM. Moreover, no long-term data were provided for most cases. This is important, since titers may fall below cut-off temporarily following treatment with steroids, plasma exchange, or immunosuppressants (or even spontaneously) and rise again at a later disease stage; accordingly, (transient) seroconversion has also been observed in a few patients with relapsing disease [2, 14, 33]. It would therefore be challenging to base long-term treatment decisions solely on whether MOG-IgG disappears or not after a first attack. If long-term treatment with immunosuppressants or oral steroids is abandoned by reason of conversion to seronegativity, close monitoring of the patient’s MOG-IgG serostatus is highly recommended to confirm seronegativity in the long-term course. Before making a diagnosis of “monophasic” MOG-EM and thus a decision against long-term treatment, one should also take into account that the interval between first and second attack in relapsing MOG-EM varies considerably among patients, with the second clinical attack occurring only after an interval of several years in some cases [3].

## Diagnostic criteria for MOG-EM

There is an unmet need for diagnostic criteria for MOG-EM. However, no specific clinical or radiological findings (except for the general requirement of a demyelinating CNS lesion) have yet been identified that are present in all MOG-IgG-positive patients and which would thus represent a diagnostic sine qua non. A lack of Dawson’s finger lesions and ovoid/round lesions on brain MRI have been proposed to be typical for MOG-EM, but this awaits confirmation in independent and larger cohorts [36, 37]. We propose that for the time being MOG-EM should be diagnosed in all patients who meet all of the following criteria:

1. Monophasic or relapsing acute ON, myelitis, brainstem encephalitis, or encephalitis, or any combination of these syndromes
2. MRI or electrophysiological (visual evoked potentials in patients with isolated ON) findings compatible with CNS demyelination
3. Seropositivity for MOG-IgG as detected by means of a cell-based assay employing full-length human MOG as target antigen

In patients with conditions considered “red flags” as defined in Table 4 and in whom MOG-IgG has not yet been confirmed in a second (and third if necessary), methodologically different cell-based assay, a diagnosis of “possible MOG-EM” should be made, especially in the context of clinical studies and treatment trials.

## Limitations and caveats

It is a limitation that all recommendations given here are necessarily based on expert consensus, owing to a lack of systematic and prospective studies. Moreover, as a general caveat, it should be stressed that before a diagnosis of MOG-EM is made, all available information, including clinical, radiological, electrophysiological, and laboratory data, need to be taken into account, and differential diagnoses, some of which are listed in Table 4, need to be excluded. Most of the information given in a previous consensus paper on differential diagnosis in MS [38] is also pertinent to MOG-EM. Finally, while the criteria proposed here can certainly help in identifying pediatric patients at high risk of being positive for MOG-IgG, they are primarily intended for use in adults and adolescents. Indications for MOG-IgG testing in children do not need to be as rigorous as in adults, since MOG-IgG is thought to be much more common in children with acquired demyelinating disease (up to 70% depending on age) than in their adult counterparts (≤ 1% in Western countries; probably ≤ 5% in Japan and other Asian countries because of lower MS prevalence). In consequence, the risk of an unfavorable ratio of FP to TP results outlined above is lower in children. While ADEM is the predominant clinical association in young children, in older children with MOG antibodies there is a shift towards presentation with ON, myelitis, and/or brainstem symptoms [11].

## Conclusion

Here, we give for the first time indications for MOG-IgG testing and propose preliminary criteria for the diagnosis of MOG-EM. While we believe that our recommendations are highly timely considering the large numbers of patients currently being tested, we are well aware that they reflect current knowledge in an evolving field and may need to be adjusted when new clinical and paraclinical data emerge and novel and optimized assays become available.

## Abbreviations

ADEM: Acute disseminated encephalomyelitis
ADEM-ON: ADEM with recurrent ON
AQP4: Aquaporin-4
CNS: Central nervous system
CRION: Chronic relapsing inflammatory optic neuropathy
CSF: Cerebrospinal fluid
EM: Encephalomyelitis
Gd: Gadolinium
IA: Immunoadsorption
IgA: Immunoglobulin A
IgG: Immunoglobulin G
IgM: Immunoglobulin M
IVMP: Intravenous methylprednisolone
LETM: Longitudinally extensive transverse myelitis
MOG: Myelin oligodendrocyte glycoprotein
MRI: Magnetic resonance imaging
MRZ: Measles, rubella and zoster virus
MS: Multiple sclerosis
NMO: Neuromyelitis optica
NMOSD: NMO spectrum disorder
OCB: Oligoclonal IgG bands
ON: Optic neuritis
PEX: Plasma exchange
PML: Progressive multifocal leukoencephalopathy
PPMS: Primary progressive MS
PRES: Posterior reversible encephalopathy syndrome
RRMS: Relapsing-remitting MS
SPMS: Secondary progressive MS
VEP: Visual evoked potentials
VS: Vertebral segments
WCC: White cell count
